# Pleural and Pericardiac Effusion as a Complication of Properly Placed Umbilical Venous Catheter

**DOI:** 10.21699/jns.v6i2.508

**Published:** 2017-04-15

**Authors:** Sezin Unal, Ilter Arifoglu, Istemi Han Celik, Osman Yilmaz, Ahmet Yagmur Bas, Nihal Demirel

**Affiliations:** 1 Department of Neonatology, University of Health Sciences, Etlik Zubeyde Hanim Women’s Health Teaching and Research Hospital, Ankara, Turkey; 2 Department of Pediatric Cardiology, University of Health Sciences, Etlik Zubeyde Hanim Women’s Health Teaching and Research Hospital, Ankara, Turkey

**Keywords:** Neonate, Umbilical venous catheter, Pleural effusion, Pericardial effusion

## Abstract

Pleural and pericardial effusions are extremely rare complications of umbilical venous catheterization in newborns. A preterm male infant weighing 850g, with insertion of an umbilical venous catheter (UVC) developed massive right pleural and pericardial effusions. The position of catheter tip was verified by chest radiography and echocardiography. The effusions were drained by thoracentesis and pericardiocentesis without complication, and were biochemically similar as total parenteral infusion which infused through catheter.

## Case Report:

**Figure 1:**
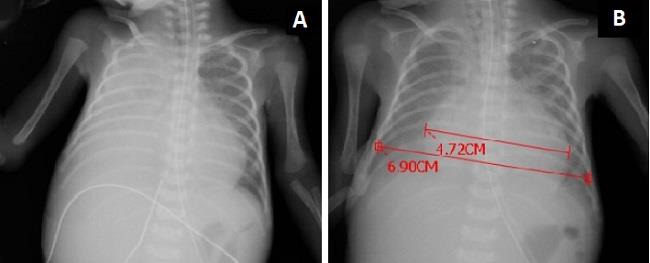
A. Chest radiograph shows right-sided pleural effusion. Note the catheter tip was in correct position. B. Chest radiograph after right thoracentesis. Catheter was removed. Lung fields were clear. Cardiothoracic index increased (0.66).

A preterm male infant (865g, 29 weeks of gestation), delivered by cesarean section, presented with non-reassuring fetal status. The APGAR score at fifth minute was eight. On admission, he presented clinical and radiological features of respiratory distress syndrome, and was placed on nasal intermittent positive pressure ventilation. Penicillin-G and gentamycin were started as empirical treatment. The UVC (4F, VYGON, France) was placed without complication, the location of catheter tip was verified by chest radiograph, and the total parenteral nutrition was started on the day of admission. Blood culture was sterile, therefore antibiotic treatment was stopped at 72nd hour. The infant remained stable until he got intubated for gradual increase in respiratory distress on the 4th day. Chest radiograph (Fig. 1A) and right lateral decubitus view showed massive right pleural effusion. The echocardiography revealed the correct position of catheter tip while TPN (osmolarity: 875 mOsm/L) was being infused. Immediately after the diagnosis of effusion was made, the UVC was removed. Needle thoracentesis was performed and it was noticed that the drained pleural fluid was similar to the TPN infusion in appearance which was also supported by biochemical analyses. The volume of pleural fluid drainage was 20 ml. Control chest radiograph revealed clear lung fields besides an apparent pericardial effusion with increased cardiothoracic index (Fig.1B). His mean arterial pressure (23 mmHg) was low, capillary refill time was prolonged to 5 seconds. Following echocardiography that established pericardial effusion, 25 ml of fluid similar to TPN infusion was drained by needle pericardiocentesis via a 24-gauge intravenous cannula. Cardiopulmonary status was stabilized after drainage. Post-procedure chest radiograph showed clear parenchymal lung fields and normal cardiac silhouette. Absence of pericardial fluid was also confirmed with echocardiography. Trace amount of left pleural effusion was observed during echocardiography, however it resolved without intervention. The infant remained intubated for three days and received TPN for 11 days. He was discharged without oxygen at 95th days of life. 

## DISCUSSION

The UVCs have considerable benefits in preterms though complications might arise in up to 20% of patients. The most common complication is the malposition. On the other hand, pleural or pericardial effusions rarely occur.[1] Infusion fluid may leak into the pleural/pericardial spaces by either direct perforation of the myocardium or endothelial damage and subsequent transmural necrosis due to repetitive hitting of catheter tip to myocardium with each heartbeat.[2]. Reported cases emphasized that proper positioning of UVCs does not guarantee the prevention of complications as happened in our case.[3,4] The mechanism to explain the properly placed catheter to cause effusions may be the repetitive hitting of catheter tip to the intrapericardial portion of inferior vena cava as Onal et al previously demonstrated perforation of inferior vena cava by linogram.[5] Traen el al suggested the hyperosmolar TPN solutions as the cause pleural/pericardial effusions due to osmotic injury.[6] In the presented case the osmolarity of TPN solution was 875 mOsm/L. Even though, TPN solutions with osmolarity up to 900 mOsm/L are accepted as suitable for peripheral administration, some data suggest a limit of 500 – 700 mOsm/L.[7] Thus, the osmolarity may contribute to the vascular damage process in similar way as extravasation and infiltration injuries that occur during peripheral TPN infusions. 

The verification of catheter tip is important both at placement and during follow-up. Even though we excluded malposition of catheter tip by echocardiography, the presented case showed that proper positioning does not avoid complications. It is remarkable that, isolated pericardial effusions were mostly reported due to mal-positioned UVCs, whereas pleural effusions were mostly reported with properly placed UVCs.[8] The case we presented also supported this observation. Hence, pleural effusions were generally diagnosed when respiratory distress symptoms became obvious, clinicians must be aware of that risk, and should not underestimate the newly produced respiratory distress symptoms in infants with proper placed UVCs.

## Footnotes

**Source of Support:** None

**Conflict of Interest:** None
